# Attachment in Couples Coping with Cancer: Associations with Observed Communication and Long-Term Health

**DOI:** 10.3390/ijerph20075249

**Published:** 2023-03-24

**Authors:** Katherine Ramos, Karena Leo, Laura S. Porter, Joan M. Romano, Brian R. W. Baucom, Shelby L. Langer

**Affiliations:** 1 Department of Psychiatry and Behavioral Sciences, Duke University Medical Center, Durham, NC 27705, USA; 2 Center for the Study of Human Aging and Development, Duke University, Durham, NC 27705, USA; 3 Department of Population Health Sciences, Duke University Medical Center, Durham, NC 27705, USA; 4 Department of Psychiatry and Behavioral Sciences, University of Washington, Seattle, WA 98195, USA; 5 Department of Psychology, University of Utah, Salt Lake City, UT 84112, USA; 6 Center for Health Promotion and Disease Prevention, Edson College of Nursing and Health Innovation, Arizona State University, Phoenix, AZ 85004, USA

**Keywords:** cancer, couple communication, attachment, dyadic coping, physical well-being

## Abstract

Cancer poses a threat to well-being that may activate the attachment system and influence interpersonal dynamics, such as communication. Research indicates that avoidant and anxious attachment, as well as communication, are independently associated with poorer psychosocial well-being, yet studies examining links between attachment, communication, and long-term physical well-being are lacking. We examined (a) associations between patient and partner attachment (measured with the adult attachment scale [AAS-Revised]) and observed communication (across affect [the Relational Affective Topography System (RATS) coding system] and behavior [the Asymmetric Behavior Coding System (ABCS) coding system]) and (b) the extent to which attachment and communication independently predicted long-term physical well-being (measured by the Functional Assessment of Cancer Therapy-General Population [FACT-GP]). Participants were 134 couples [mean age 53.9 (*SD* = 13.4), 86.2% Caucasian, 66% of patients, 36% of partners female]. Patient participants had either breast, colorectal, or lung cancer. Couples individually completed self-report measures of attachment (baseline) and physical well-being (baseline and 4, 8, and 12 months later). At baseline, couples engaged in a 15 min videorecorded cancer-related conversation coded for communication behavior and affective expression. Patients and partners with higher anxious and avoidant attachment exhibited more negative affect and negative approach behaviors. A greater avoidant attachment was associated with less positive affective expression. Attachment insecurity and affective expression were prospectively linked with physical well-being. Findings indicate that attachment is associated with overt communication behaviors and that insecure attachment and affective expression may be risk factors for poorer health outcomes.

## 1. Introduction

Cancer is a life-altering event for patients and caregivers that presents emotional, social, and physical challenges. These challenges often disrupt the quality of life for both patients and their caregiving spouses/intimate partners [1,2,3]. The psychological effects of cancer on a patient may be strongly influenced by their interpersonal context and, particularly, their interactions with their partner. Attachment theory [4,5] can provide a heuristic framework for understanding the psychosocial impact of cancer on both patients and partners and its influence on the ways in which people convey their thoughts and feelings when talking about cancer. It may also provide an explanatory model for understanding and predicting variability across couples in their psychological responses to cancer. 

Attachment is theorized to be influenced by the early parent–child interactions that shape a sense of security and protection during periods of heightened distress or perceived threat. In turn, these interactions influence life-long patterns of emotional regulation and response to stress, particularly whether individuals seek closeness to significant others and see them as a source of comfort or support in times of stress. The consistent or inconsistent support of primary attachment figures (i.e., parents/caregivers) to their child in need impacts the child’s affective, behavioral, and cognitive responses to distressing life events [6]. The premise holds that these early experiences lead to different attachment styles [4,5] (i.e., patterns of expectations, feelings, and behaviors) that inform emotion regulation capacities and psychological adjustment during distressing events through adulthood. 

Adult attachment is commonly measured along dimensions of attachment avoidance and anxiety [7]. Individuals with a predominantly avoidant attachment style are uncomfortable with intimacy and emotional closeness. Conversely, those with a predominantly anxious attachment style tend to be preoccupied with proximity to attachment figures, seeking to maintain emotional closeness. Individuals with a secure attachment style (low in dimensions of avoidance and anxiety) can self-soothe and regulate emotions appropriately without displaying behaviors of overdependence or distancing in times of need. Unlike parent–child relationships, in adult relationships, caregiving processes are highly interrelated. For example, dyad members (i.e., patient and partner) may rely on each other to satisfy attachment needs. Thus, the attachment styles of both members may influence how they respectively regulate their emotions and behaviors and interact with each other. Unsurprisingly, the inability to self-regulate during periods of illness, such as cancer, can lead to heightened distress and relationship dysfunction for both the patient and partner. 

**Attachment, Psychosocial, and Physical Well-Being.** Among cancer patients, patterns of attachment insecurity have been associated with poorer physical health, physical well-being, and psychosocial adjustment [8,9,10,11,12,13,14]. Studies have demonstrated that patients higher in anxious attachment showed decreases in immune functioning [7,15,16]. They also reported greater depression, hopelessness/helplessness, anxious preoccupation, and lower social well-being [11,12]. Patients scoring high in avoidant attachment reported greater depression and marital dissatisfaction, and lower quality of life [17,18,19,20,21,22,23]. In addition, studies have demonstrated that patients scoring higher in both attachment anxiety and avoidance reported greater cancer-related distress and lower well-being [18]. 

Attachment also affects how caregiving partners cope with their loved one’s cancer diagnosis, and the quality of care and support they offer to their loved one. Among spousal caregivers, those with an anxious attachment style reported higher anxiety, poorer life satisfaction, and higher depression compared to securely attached caregivers [18,24]. Those scoring high in avoidant attachment reported higher levels of caregiver strain and anger and lower levels of marital quality and life satisfaction [17,18,25]. 

Despite the relevance of attachment in relationships, few studies have examined attachment in both patients and caregivers. Findings suggest that in dyads in which both members are secure, both patients and caregivers report better adjustment compared to dyads in which both are insecure [18,19,20]. In dyads in which one member is secure and the other is insecure, scores fall somewhere in between on measures of individual and relationship adjustment [18,19,21,22]. 

**Attachment and Communication.** Despite strong evidence that attachment styles are linked to adjustment to cancer, there has been little attention to how attachment may influence patient–partner communication about cancer. Research conducted with romantic partners outside the context of cancer suggests that insecure attachment interferes with effective communication behaviors (e.g., defensiveness, blame) during marital conflicts [26], but few studies have examined this in couples coping with cancer or using observed behavior. Findings from a recent systematic review on cancer-related communication among couples suggest that avoiding communication negatively impacts individual and interpersonal well-being; and the extent to which couples share (i.e., disclose) is only beneficial within context (e.g., when a partner is responsive to the disclosure) [27]. What couples share and how responsive a partner is to the disclosure may be due to individual differences. We believe that attachment style is one such example of an individual difference (e.g., during disclosures one’s attachment style may contribute to either seeking support/closeness or avoidance when managing difficult emotions). However, the reviewed studies did not examine associations between communication and attachment with long-term health. This novel line of investigation is one we seek to address in this study. In a prior cross-sectional study based on the same sample as the current study [13], our research group examined the concurrent relationship between attachment and self-reported communication about cancer, as well as physical well-being at baseline. We found that anxious attachment in patients and spouses was positively associated with both self-reported emotional disclosure and holding back of thoughts and feelings about cancer and its treatment and was negatively associated with the physical well-being of both members of the couple. Avoidant attachment in patients and spouses was directly positively associated with holding back and inversely associated with physical well-being. Path analyses indicated that disclosure and holding back mediated the attachment–physical well-being relationship both within persons and across partners. These findings lend support to the idea that communication behaviors provide a linkage between attachment characteristics and well-being, but these findings are limited by their reliance on self-report and the cross-sectional nature of the analyses. 

Observational coding of couples’ interactions may be a potentially valuable approach to studying communication among couples coping with cancer, as self-report of communication behaviors may not always be accurate or fully represent actual behavior [28,29]. Observational assessment of communication provides unique opportunities to identify important and specific communication behaviors, including affective expressions, that may be associated with dimensions of attachment, and that may also be predictive of better adjustment. Identifying these behaviors can inform clinical interventions that account for the couple’s attachment needs and improve their adaptation to cancer. One approach, for example, is to use communication models, such as the Valence Affective Communication (VAC) model, which theorizes how communication can be categorized across affective expression and communication behavior [28,29]. For example, it is plausible that disclosure of distressing thoughts and feelings may be more challenging for patients and partners higher in attachment avoidance or anxiety. They may potentially benefit from coaching in how and what to disclose while using positive joining emotions (e.g., appreciation of the other) or soft negative emotions (e.g., sadness or fearfulness) rather than hard negative affective expressions such as anger or frustration. To our knowledge, no previous study has examined associations between attachment and observationally derived measures of couple communication in cancer.

**Current study.** For this study, we aimed to describe the communication correlates of attachment as observed from recorded couple conversations about cancer, including observed communication behavior and affective expression derived from the Valence Affective Communication Model (VAC [28,29]). We also examined the extent to which attachment dimensions, affective expression, and communication behaviors independently predicted physical well-being over time. 

## 2. Materials and Methods

Patient and caregiver participants were enrolled in a larger observational study examining couple’s communication in cancer (NCI: R01CA201179). Full protocol details can be found elsewhere [30]. Recruitment took place at the Duke Cancer Institute in Durham, NC, and at the Seattle Cancer Care Alliance (SCCA), now the Fred Hutchinson Cancer Center in Seattle, WA. This study was approved by the institutional review boards of Duke University and Arizona State University, with agreements in place allowing the University of Washington and Fred Hutchinson Cancer Center to rely on ASU. For this sample, the timeframe for recruitment was from May (2017)–March (2019).

Following a review of medical records by our study staff, patients who were identified as meeting initial medical inclusion criteria were sent a study brochure and letter signed by their primary oncologist introducing the study [30]. The letter also informed prospective participants that they would be contacted by a research staff member [30]. Opt-out instructions for non-participation were also included in the letter. Patients who were contacted and considered eligible were given a description of the study objectives [30]. If the patient decided to participate, the research staff member then obtained permission to speak with the partner to gauge interest [30]. Additional recruitment details can be found elsewhere [30]. Eligible participants completed a written informed consent. Inclusion criteria for patients were stage II–IV breast, lung, colon, or rectal cancer. Patients were within 2 years of diagnosis of their current cancer stage; currently receiving or having received chemotherapy and/or hormone therapy. Patients also needed to have a life expectancy of at least 6 months (determined by their oncologist). Patients needed to be married or in a committed, cohabiting relationship. Both patients and partners had to be at least 18 years old or older and be able to speak and comprehend English.

Participants completed a baseline assessment which included self-report measures and a 15 min cancer-related conversation which was videorecorded for subsequent coding. They completed self-report measures at 4-, 8-, and 12-month follow-ups [30]. 

### 2.1. Measures

#### 2.1.1. Self-Report Measures

Attachment. The 18-item AAS-Revised [31,32] asks respondents to rate their feelings about their romantic relationships on a five-point Likert-type scale (“Not at all characteristic of me” to “Very characteristic of me”). Higher scores indicate greater attachment insecurity. For our study, we computed scores for attachment anxiety and attachment avoidance. Sample items include “I often worry that other people won’t want to stay with me” (attachment anxiety) and “I find it difficult to allow myself to depend on others” (attachment avoidance). This measure was completed at baseline only. In this study, Cronbach alphas for the attachment anxiety scale were α = 0.91 (patients) and α = 0.85 (partners); for the attachment avoidance scale they were α = 0.84 (patients) and α = 0.85 (partners).

Physical well-being. Physical well-being (PWB) was assessed using the 7-item physical well-being subscale from the Functional Assessment of Cancer Therapy scale (FACT-GP) [33], a widely used and well-validated measure of the quality of life. Items include “I have pain” and “I feel ill” (reverse-scored). Participants rate each item on a five-point response scale ranging from 0 (“not at all”) to 4 (“very much”). Higher scores indicate better well-being. Participants completed this measure at four timepoints, baseline, 4, 8, and 12 months. Cronbach alphas for our study are as follows: Patients-Time 1 α = 0.83, Time 2 α = 0.88, Time 3 α = 0.86, Time 4 α = 0.87; Partners-Time 1 α = 0.69, Time 2 α = 0.82, Time 3 α = 0.72, and Time 4 α = 0.76.

#### 2.1.2. Observational Measures

We utilized two observational coding systems based on the Valence Affective Communication model to capture communication in couples. The VAC model [28,29] posits that dyadic exchanges can be conceptualized as comprising communication behavior and affective expression and theorizes that these two constructs of communication can be further categorized in terms of valence (positive–negative) and a speaker’s goals (joining–individuating) [28,29]. For the latter, affective expression and communication behavior that are joining in nature tend to prioritize the needs of the relationship, whereas individuating communication promotes the individual’s needs over the relationship [28,29]. 

Communication behavior. The Asymmetric Behavior Coding System (ABCS [28,29]) is designed to measure communication behavior. This system groups communication behavior into broad categories of negative and positive valence, which are then further delineated into those that are joining versus individuating, resulting in four categories. Coders are asked to rate each communication behavior from the following categories on a scale of 1 (no behavior) to 7 (high levels of behavior): (a) positive approach (maintaining/deepening, disclosure, validation, collaboration, intimacy building, justification), (b) positive avoidance (accommodation, tough love, minimization, reassurance), (c) negative approach (blame, belligerence, contempt, dominance, emotional protests, defensiveness, pressure for change), and (d) negative avoidance (withdrawal, avoidance, stonewalling, submit, controlling the conversation). Coders underwent approximately six weeks of training and attended weekly meetings with the lead trainer to maintain inter-rater reliabilities and resolve discrepancies among coders. 

Affective expression. The Relational Affective Topography System (RATS [28,29]) is an observational coding system designed to measure affective expression. In this system, affective expression is categorized in terms of valence: flat (boredom and indifference), positive, and negative. The positive and negative affective expressions are further defined into categories of joining versus individuating goals: positive joining (warmth, appreciation, kindness), positive individuating (happiness, enthusiasm, amusement, satisfaction), hard negative (anger, disgust, frustration, outrage), and soft negative (sadness, fearfulness, loneliness, guilt, vulnerability). The RATS uses a top-down approach, where coders first assess whether flat, positive, or negative affective expressions are present. Coders then rate each specific affective expression in the indicated category on a scale of 0 (no affective expression) to 7 (high levels of affective expression). For this study, coders went through three weeks of training and attended a coding meeting with the lead trainer weekly to reduce rating discrepancies and increase reliabilities.

### 2.2. Analytic Plan

Aim 1: To examine associations between attachment and observationally coded behavior. These associations were analyzed using two-level mixed-effect models in Stata Version 17 [34]; multi-level modeling was indicated due to the nested structure of the data. Separate models were estimated using each affective expression and communication behavior as dependent variables with attachment variables (anxious or avoidant) and role (patient vs. partner), and the interaction between the two as independent variables in each model. 

Aim 2: To test associations between attachment and communication with physical well-being. Three-level mixed-effect models were estimated in Stata Version 17 [34] to (1) examine the associations between attachment (avoidant and anxious) and physical well-being across time (i.e., baseline, 4, 8, and 12 months) and (2) to examine the associations between communication (observed affective expression and communication behavior) and physical well-being over time. To understand the associations between attachment and physical well-being, two separate models were estimated with physical well-being as the dependent variable, and main effects for and interactions between attachment variables (anxious or avoidant), role (patient vs. partner), and time as independent variables. To examine the associations between communication and physical well-being over time, separate models were estimated where physical well-being was regressed onto main effects for and interactions between communication behavior or affective expression, role, and time. Separate models were estimated for each category of affective expression and communication behavior, resulting in eight models. 

## 3. Results

The sample consisted of 268 patients and partners (i.e., 134 couples). Patients were diagnosed with breast (46%), colorectal (42%), or lung (12%) cancer. Participants’ mean age was 53.9 (*SD* = 13.4). A total of 86.2% were Caucasian. A total of 66% of patients and 36% of partners were female. 

Inter-rater reliabilities for the RATS coding system were α = 0.87 for hard negative affective expression, α = 0.87 for soft negative affective expression, α = 0.90 for positive joining affective expression, and α = 0.93 for positive individuating expression. Inter-rater reliabilities for the ABCS coding system were α = 0.82 for positive avoidance, α = 0.94 for positive approach, α = 0.94 for negative avoidance, and α = 0.99 for negative approach. Descriptive statistics and correlations between key study variables are summarized in Table 1 and Table 2 for patients and partners, respectively. Of note, across the four timepoints in our study, at Time 1 we had 134 couples, at Time 2 we had 103 couples, at Time 3 we had 96 couples, and at Time 4 we had 89 couples. There were a few data points missing for three partners; the change of N over time is summarized accordingly in the last row of Table 2. The other variables collected at baseline from our study were among the 134 couples. 

### 3.1. Aim 1 Findings

**Anxious attachment, affective expression, and communication behavior.** Results showed that higher levels of anxious attachment were associated with higher levels of hard negative affect (B = 0.12, *p* = 0.006) and soft negative affect (B = 0.15, *p* = 0.003) for both patients and partners. Additionally, anxious attachment was associated with increases in negative approach behavior (B = 0.11, *p* = 0.006) for both patients and partners. See Table 3.

**Avoidant attachment, affective expression, and communication behavior.** For both patients and partners, avoidant attachment was inversely associated with positive joining affect (B = −0.24, *p* = 0.001) and positive individuating affect (B = −0.17, *p* = 0.013), and positively associated with hard negative affect (B = 0.19, *p* = 0.002). Higher avoidant attachment was also positively associated with negative approach behavior for both patients and partners (B = 0.16, *p* = 0.004). Of note, the analysis examining avoidant attachment in predicting positive avoidance could not be run due to low variance in the independent variable. Please see Table 4. 

**Differences in affective expression and communication behavior by role**. A pattern of mean level differences in affective expression and communication behavior between patients and partners emerged across the separate models estimated. Results showed that partners exhibited less positive individuating and soft negative affective expressions than patients. In addition, partners exhibited more positive and negative avoidance behaviors compared to patients. Please see Table 3 and Table 4 for the complete results. 

### 3.2. Aim 2 Findings 

**Differences in physical well-being by role.** Across the models, there was a mean level difference in physical well-being between patients and partners, such that partners reported better physical well-being compared to patients. Please see Table 5, Table 6 and Table 7 for the results. 

**Time, role, and physical well-being.** A significant interaction between role and time emerged in predicting physical well-being emerged across all models. Decomposition of the interaction showed that there was a significant effect of time on patients’ well-being, such that well-being improved over time for patients (B = 0.07, *p* = 0.000) but not for partners (B = −0.04, *p* = 0.063). Please see Table 5 and Table 6 for the results.

**Table 5:** Three-level mixed effect models of physical well-being, time, and attachment for patients and partners.

	Physical Well-Being				Physical Well-Being		
	*B*	*SE B*	95% CI		*B*	*SE B*	95% CI
Intercept	2.85 ***	0.06	[2.75, 2.96]	Intercept	2.83 ***	0.05	[2.72, 2.93]
Anxious Attachment	−0.25 ***	0.06	[−0.36, −0.13]	Avoidant Attachment	−0.44 ***	0.08	[−0.60, −0.28]
Role	0.63 ***	0.07	[0.49, 0.77]	Role	0.68 ***	0.07	[0.54, 0.81]
Role × Anxious Attachment	0.06	0.09	[−0.12, 0.23]	Role × Avoidant Attachment	0.24 *	0.11	[0.02, 0.46]
Time	0.07 ***	0.02	[0.03, 0.11]	Time	0.07 ***	0.02	[0.04, 0.11]
Anxious Attachment × Time	0.03	0.02	[−0.01, 0.08]	Avoidant Attachment × Time	0.06	0.03	[−0.01, 0.12]
Role × Time	−0.11 ***	0.03	[−0.16, −0.05]	Role × Time	−0.11 ***	0.03	[−0.16, −0.06]
Role × Anxious Attachment × Time	−0.02	0.03	[−0.09, 0.05]	Role × Avoidant Attachment × Time	−0.05	0.04	[−0.13, 0.04]
				Decomposition of Interactions			
				Role x Avoidant Attachment (partner)	−0.20 *	0.08	[−0.36, −0.04]
				Role × Time (patient)	0.07 ***	0.02	[0.04, 0.11]
				Role × Time (partner)	−0.04	0.02	[−0.08. 0.00]

*Note.* The left side of the table represents the model with anxious attachment, while the right side of the table represents the model with avoidant attachment. Attachment variables were grand-centered. For role, patient was coded as 0 and partner was coded as 1. * *p* < 0.05. *** *p* < 0.001.

**Attachment and physical well-being.** Results showed that on average, patients and partners higher in anxious attachment reported lower physical well-being (B = −0.25, *p* = 0.000). For avoidant attachment, a significant interaction emerged between role and avoidant attachment (B = 0.24, *p* = 0.034). Decomposition of the interaction suggested that avoidant attachment was associated with lower well-being for both patients (B = −0.44, *p* = 0.000) and partners (B = −0.20, *p* = 0.013); however, there was a larger effect of avoidant attachment on patients’ physical well-being as compared to partners. All effects were evident at baseline assessment and maintained over time. Please see Table 5 for the results.

**Affective expression and physical well-being.** Positive individuating (e.g., happiness, enthusiasm), soft negative (e.g., fear, sadness), and hard negative (e.g., anger, frustration) affective expressions were significantly associated with physical well-being (Table 6). A significant interaction emerged between role and positive individuating affect in predicting physical well-being (B = −0.29, *p* = 0.007). Decomposition of the interaction showed that on average, positive individuating affective expression was associated with higher levels of well-being for patients (B = 0.19, *p* = 0.022), but not for partners (B = −0.10, *p* = 0.210). For soft negative affect, a significant interaction emerged between role and soft negative affect in predicting physical well-being (B = 0.27, *p* = 0.05). Decomposition of the interaction showed that on average, expression of soft negative affect was associated with lower levels of physical well-being for patients (B = −0.30, *p* = 0.000), but not for partners (B = −0.03, *p* = 0.824). All effects were evident at baseline assessment and maintained over time.

**Table 6:** Three-level mixed effect models of physical well-being, time, and affective expression for patients and partners.

	Physical Well-Being				Physical Well-Being		
	*B*	*SE B*	95% CI		*B*	*SE B*	95% CI
Intercept	2.84 ***	0.06	[2.73, 2.95]	Intercept	2.83 ***	0.06	[2.72, 2.94]
Positive Joining	0.07	0.09	[−0.10, 0.25]	Positive Individuating	0.19 *	0.08	[0.03. 0.35]
Role	0.66 ***	0.07	[0.52, 0.80]	Role	0.67 ***	0.07	[0.53, 0.81]
Role × Positive Joining	−0.06	0.12	[−0.31, 0.18]	Role × Positive Individuating	−0.29 **	0.11	[−0.50, −0.08]
Time	0.07 ***	0.02	[0.03, 0.11]	Time	0.07 ***	0.02	[0.03, 0.11]
Positive Joining × Time	0.03	0.03	[−0.03, 0.09]	Positive Individuating × Time	−0.01	0.03	[−0.07, 0.05]
Role × Time	−0.11 ***	0.03	[−0.16, −0.05]	Role × Time	−0.11 ***	0.03	[−0.16, −0.05]
Role × Positive Joining × Time	−0.05	0.05	[−0.14, 0.04]	Role × Positive Individuating × Time	0.03	0.04	[−0.05, 0.12]
				Decomposition of interactions			
				Role x Positive Individuating (partner)	−0.10	0.08	[−0.26, 0.06]
	Physical Well-being				Physical Well-being		
	*B*	*SE B*	95% CI		*B*	*SE B*	95% CI
Intercept	2.84 ***	0.06	[2.73, 2.95]	Intercept	2.89 ***	0.06	[2.78, 3.00]
Hard Negative	−0.45 ***	0.11	[−0.68, −0.23]	Soft Negative	−0.30 ***	0.08	[−0.46, −0.13]
Role	0.66 ***	0.07	[0.52, 0.80]	Role	0.61 ***	0.07	[0.46, 0.75]
Role × Hard Negative	0.47 **	0.16	[0.16, 0.77]	Role × Soft Negative	0.27 *	0.14	[0.00, 0.54]
Time	0.07 ***	0.02	[0.04, 0.11]	Time	0.07 **	0.02	[0.03, 0.11]
Hard Negative × Time	0.13 **	0.05	[0.03, 0.23]	Soft Negative × Time	0.01	0.03	[−0.05, 0.07]
Role × Time	−0.11 ***	0.03	[−0.17, −0.06]	Role × Time	−0.11 ***	0.03	[−0.17, −0.05]
Role × Hard Negative x Time	−0.15 *	0.07	[−0.28, −0.02]	Role × Soft Negative × Time	−0.04	0.06	[−0.15, 0.07]
Decomposition of Interactions				Decomposition of Interactions			
Intercept (partner)	3.50 ***	0.06	[3.39, 3.61]	Role × Soft Negative (partner)	−0.03	0.11	[−0.25, 0.20]
Role × Hard Negative (partner)	0.01	0.11	[−0.20, 0.23]				
Hard negative affect on change in physical well-being over time (patient)	0.21 ***	0.06	[0.10, 0.32]				
Hard negative affect on change in physical well-being over time (partner)	−0.06	0.05	[−0.15, 0.04]				

*Note.* The four quadrants of the table represent the four different types of affective expression. Affective expression variables were grand-centered. For role, patient was coded as 0 and partner was coded as 1. * *p* < 0.05. ** *p* < 0.01. *** *p* < 0.001.

Lastly, for hard negative affect, a two-way interaction emerged between role and hard negative affect (B = 0.47, *p* = 0.003) and hard negative affect and time (B = 0.13, *p* = 0.009). There was also a significant three-way interaction between role, hard negative affect, and time (B = −0.15, *p* = 0.022). These associations indicate that not only is hard affect associated with differences in physical well-being at the baseline assessment but also that hard affect is associated with the rate of change in physical well-being over time. More specifically, the decomposition of the interactions suggests that higher levels of hard negative affect are associated with greater increases in physical well-being over time for patients (B = 0.21, *p* = 0.000), whereas hard negative affect is not associated with the rate of change in physical well-being for partners (B = −0.06, *p* = 0.260). Please see Table 6 for the complete results.

**Communication behavior and physical well-being.** There were no significant effects of the different categories of communication behavior on physical well-being for either patients or partners. Please see Table 7 for the results.

**Table 7:** Three-level mixed effect models of physical well-being, time, and communication behavior for patients and partners.

	Physical Well-Being				Physical Well-Being		
	*B*	*SE B*	95% CI		*B*	*SE B*	95% CI
Intercept	2.84 ***	0.06	[2.73, 2.95]	Intercept	2.84 ***	0.06	[2.73, 2.96]
Positive Approach	0.01	0.11	[−0.21, 0.23]	Positive Avoidance	0.04	0.17	[−0.29, 0.37]
Role	0.66 ***	0.07	[0.52, 0.80]	Role	0.66 ***	0.07	[0.52. 0.80]
Role × Positive Approach	−0.08	0.16	[−0.40, 0.24]	Role × Positive Avoidance	0.00	0.21	[−0.40, 0.41]
Time	0.07 ***	0.02	[0.03, 0.11]	Time	0.07 **	0.02	[0.03, 0.11]
Positive Approach × Time	−0.02	0.04	[−0.09, 0.05]	Positive Avoidance × Time	−0.03	0.06	[−0.14, 0.08]
Role × Time	−0.11 ***	0.03	[−0.16, −0.06]	Role × Time	−0.10 ***	0.03	[−0.16, −0.05]
Role × Positive Approach × Time	−0.01	0.06	[−0.13, 0.10]	Role × Positive Avoidance x Time	−0.04	0.07	[−0.18, 0.11]
	Physical Well-being				Physical Well-being		
	*B*	*SE B*	95% CI		*B*	*SE B*	95% CI
Intercept	2.84 ***	0.06	[2.73, 2.95]	Intercept	2.84 ***	0.06	[2.73, 2.96]
Negative Approach	−0.14	0.12	[−0.38, 0.10]	Negative Avoidance	0.04	0.09	[−0.14, 0.22]
Role	0.67 ***	0.07	[0.53, 0.81]	Role	0.66 ***	0.07	[0.52, 0.80]
Role × Negative Approach	−0.02	0.16	[−0.35, 0.30]	Role × Negative Avoidance	−0.05	0.12	[−0.28, 0.18]
Time	0.07 ***	0.02	[0.03, 0.11]	Time	0.07 ***	0.02	[0.04, 0.11]
Negative Approach × Time	0.02	0.05	[−0.06, 0.11]	Negative Avoidance × Time	0.03	0.03	[−0.03, 0.09]
Role × Time	−0.11 ***	0.03	[−0.16, −0.05]	Role × Time	−0.11 ***	0.03	[−0.17, −0.06]
Role × Negative Approach × Time	−0.01	0.06	[−0.13, 0.11]	Role x Negative Avoidance × Time	−0.05	0.04	[−0.13, 0.03]

*Note.* Communication behavior variables were grand-centered. For role, patient was coded as 0 and partner was coded as 1. ** *p* < 0.01. *** *p* < 0.001.

## 4. Discussion

Overall, our study findings suggest that attachment is associated with observationally derived measures of patient–partner communication during a cancer-related conversation. Among both patients and partners, those who reported higher levels of anxious attachment displayed more negative emotions (affect) and negative approach behaviors during the conversation. Anxious attachment is characterized by fear of abandonment and the need for validation. In this study, patients and partners who scored higher in attachment anxiety also demonstrated more use of negative, potentially maladaptive communication strategies, including more use of hard and soft negative affect and communication behaviors, such as blaming or pressuring the partner. In this scenario, an anxiously attached cancer patient’s or partner’s desire to seek stability and reassurance in the relationship could be undermined by their own difficulty in regulating their emotions and the use of maladaptive communications. As such, these affective expressions and communication behaviors have the potential to shut down or inhibit how patients and partners connect in the context of cancer-related conversations. For example, an anxious partner’s behavior may result in their loved one feeling as if discussions about cancer will create highly emotional and tumultuous exchanges, which may cause distress, avoidance of communication, and lower relationship satisfaction [35]. Thus, the very attempts to elicit reassurance and support from the other member of the couple may backfire due to poor communication. 

We also found that higher levels of avoidant attachment were linked to less positive joining affect (e.g., warmth, appreciation), less positive individuating affect (satisfaction, happiness), and greater use of hard negative affect (e.g., anger, disgust), and negative approach behaviors (e.g., blame, belligerence). Given that avoidantly attached individuals are generally highly independent and uncomfortable with closeness, it is not surprising that in the context of a conversation about cancer, these individuals displayed affect and behaviors that could limit intimacy. In dealing with cancer, being partnered with an avoidantly attached person may make the individual feel disconnected from the other and unsupported, which may negatively impact their emotional and relational adjustment [8]. 

Of note, independent of patient or partner attachment style, we also found differences in patients and partners across displays of affective expressions and communication behaviors. Specifically, while discussing a cancer-related topic of their choice, partners compared to patients exhibited less positive individuating and soft negative affective expressions, and more positive and negative avoidance behaviors (e.g., accommodating, and being agreeable to the demands of the patient or withdrawing or stonewalling the conversation). Positive individuating emotions are emotions that reflect an individual’s own positive emotional state independent of the partner. Soft negative emotions are emotions that reflect and communicate distress or vulnerability. For partners of cancer patients, there may be a strong desire to be as fully supportive as possible of their loved one’s experience and, as such, the partner may overprioritize how the patient feels above expressing their own feelings and distress. It may also be the case that the task demands of discussing a cancer-related topic may have prompted the partner to focus on the patient’s experience rather than their own. 

In our examination of physical well-being over time, we found that partners reported better physical well-being compared to patients. This finding is to be expected given that one partner is living with cancer and the other is not. Interestingly, however, we found that well-being improves over time for patients only. One plausible explanation is that couples were recruited during treatment for cancer and may have been experiencing side effects that improved over time when active treatment was not taking place. Another possibility is that, over time, cancer patients learn or adopt specific strategies that enhance their perceptions of how they cope and manage their health [36,37]. However, of note is that the trajectory of physical well-being over time depended on attachment for both patients and partners. Patients and partners higher in anxious and avoidant attachment reported lower physical well-being over time, and the effect of the association between avoidant attachment and lower physical well-being was larger for patients compared to partners. Patients with cancer who avoid closeness and intimacy may inadvertently be depriving themselves of the significant health benefits that can come from establishing and maintaining meaningful social connections. For example, some studies suggest that avoidance in a relationship is associated with higher cortisol reactivity, and perhaps in the context of cancer, this may imply that the patient is more physiologically taxed than their healthy partner [38,39]. 

Lastly, analyses of associations between communication and physical well-being indicated that positive individuating expression and hard negative affective expression were associated with higher levels of well-being over time for patients but not for partners. There were no significant effects of communication behaviors on the physical well-being of patients or partners. Overall, these last set of findings highlight that affective expression and not communication behavior per se may influence patient perceptions of their physical well-being. For patients, finding opportunities to display their happiness, enthusiasm, and satisfaction, as well as normative reactions of anger and frustration in the context of cancer, may be adaptive and healthy. Moreover, it may be that patients who display and share these affective expressions are better able to understand their own emotions and feel more in control, and thus endorse better health outcomes [40,41]. Overall, these findings have potentially important clinical implications for the development of psychosocial interventions for individuals and couples coping with cancer. There are opportunities to tailor interventions based on attachment styles to enhance communication [19,21,22,42,43,44]. As reported in the previous literature [27], communication in the form of hiding concerns and negative feelings (i.e., protective buffering) is linked with negative individual and relational outcomes. Attachment may offer unique ways to approach adaptive disclosure and protective buffering in communication. One example includes helping anxiously attached patients and partners reduce maladaptive communication strategies aimed at seeking validation and support from their partners. Anxiously attached individuals, for example, may be taught to reframe negative emotions, be mindful of how much and what is disclosed, and utilize more adaptive behavioral strategies (e.g., expressing warmth, appreciation, and intimacy building) rather than maladaptive ones, such as blaming or demanding during communication exchanges. For avoidantly attached patients and partners, one approach may include helping them to communicate empathically and become more comfortable with positive affective expression while respecting their desire for privacy. They may also benefit from learning perspective-taking about how the other may feel in the context of cancer to help bolster connection and closeness. Based on these current findings, examining affective and behavioral correlates of attachment in communication can provide unique opportunities to identify intra- and interpersonal factors that affect how couples cope with cancer. 

Incorporating attachment-informed strategies into couple-based communication interventions for cancer patients and their partners has the potential to increase the efficacy of these interventions, with the goal of helping couples better support each other while coping with cancer. Our findings contribute to the larger couple’s communication in cancer literature and suggest that blanket prescriptions to either disclose, share, or even express are likely not always warranted. In particular, disclosure enacted by persons high in anxious attachment may be maladaptive, as their desire to seek closeness may come in the form of highly dysregulated emotional expression, making it difficult for the partner to listen in an effective manner. Our study is also unique in its use of objective coding to discern both the valence and function of communication behaviors and expressions (e.g., joining versus distancing), that to date has been largely missing in the research literature.

Despite our study’s strengths and potential impact on future work, there are a few limitations to note. First, although we longitudinally examined health over time our predictor variables of attachment and communication behavior were only collected at baseline (one timepoint). Further, while this study had access to a large couple dataset to study interpersonal dynamics, the sample was largely Caucasian and younger than 65 years of age. It is possible that more diverse samples and older partners in longer-term relationships might have nuanced approaches on their affect and communication that influence specific affective expressions and selection of communication behaviors. Future directions would include pursuing this investigation from data consisting of a more heterogeneous sample across the lifespan. Moreover, understanding the potential mechanisms of communication in the relationship between attachment and health over time is an important exploratory study to pursue next.

## 5. Conclusions

The proposed study addresses major gaps in couple communication and cancer research using an attachment framework. Our study results provide an initial and novel investigation of interpersonal processes and behaviors through which attachment in both cancer patients and their partners may influence communication and physical well-being. Results of the present study suggest that assessing attachment may provide additional context and information for clinicians about which affective expressions and communication behaviors to focus on during individual or couple-based interventions. In addition, these study results suggest that affective expressions may play a very nuanced and significant role in a patient’s well-being. It may be valuable to incorporate psychoeducation, self-monitoring, and techniques for enhancing affective expression in addition to communication behavior in current or future couple-based interventions to promote increased adaptation to cancer. As such, attachment theory offers a viable and novel framework for examining the affective and behavioral experiences associated with communication and outcomes in cancer patients and caregivers.

## Figures and Tables

**Table 1:** Descriptive statistics and correlations for study variables for patients.

Variable	1	2	3	4	5	6	7	8	9	10	11	12	13	14
RATS														
1. Positive Joining														
2. Positive Individuating	0.20 **													
3. Hard Negative	−0.39 **	−0.23 **												
4. Soft Negative	0.04	−0.24 **	0.12											
ABCS														
5. Negative Approach	−0.21 **	−0.22 **	0.47 **	0.15										
6. Negative Avoidance	−0.08	−0.08	0.14 *	0.06	0.22 **									
7. Positive Approach	−0.002	−0.01	0.08	0.14	0.10	−0.029 **								
8. Positive Avoidance	−0.14	0.03	0.16	0.03	0.35 **	0.08	0.10							
Attachment														
9. Anxious Attachment	−0.07	−0.15	0.25 **	0.23 **	0.24 **	0.07	0.05	0.06						
10. Avoidant Attachment	−0.26 **	−0.13	0.28 **	0.08	0.22 **	0.13	0.002	0.17	0.60 **					
Health														
11. Physical Well-being at baseline	0.05	0.21 *	−0.29 ***	−0.27 **	−0.07	0.02	−0.02	−0.03	−0.31 ***	−0.37 ***				
12. Physical Well-being at 4 months	0.05	−0.03	−0.05	−0.14	−0.02	0.12	0.04	0.05	−0.16	−0.23 **	0.66 ***			
13. Physical Well-being at 8 months	0.10	0.14	0.05	−0.29 **	0.00	0.04	0.07	−0.04	−0.15	−0.12	0.51 ***	0.49 ***		
14. Physical Well-being at 12 months	0.07	−0.07	−0.03	−0.24 *	0.03	0.07	−0.10	−0.03	−0.07	−0.16	0.50 ***	0.52 ***	0.77 ***	
Mean (SD)	0.93 (0.62)	1.11 (0.69)	0.33 (0.49)	0.83 (0.64)	1.36 (0.45)	1.91 (0.60)	3.06 (0.50)	1.26 (0.33)	2.03 (0.94)	2.47 (0.66)	2.88 (0.81)	2.88 (0.90)	3.07 (0.81)	3.19 (0.69)
n	134	134	134	134	134	134	134	134	134	134	134	103	96	89

*Note*. * *p* < 0.05. ** *p* < 0.01. *** *p* < 0.001. Data reflect responses of study variables of the patient only.

**Table 2:** Descriptive statistics and correlations for study variables for partners.

Variable	1	2	3	4	5	6	7	8	9	10	11	12	13	14
RATS														
1. Positive Joining														
2. Positive Individuating	0.12													
3. Hard Negative	−0.38 **	−0.02												
4. Soft Negative	0.07	−0.10	0.19 **											
ABCS														
5. Negative Approach	−0.26 **	−0.02	0.41 **	0.11										
6. Negative Avoidance	−0.27 **	−0.06	0.17	−0.02	0.37 **									
7. Positive Approach	0.15	−0.10	0.09	0.10	0.13	−0.11								
8. Positive Avoidance	−0.05	0.17	0.27 **	−0.08	0.17	0.09	0.20 **							
Attachment														
9. Anxious Attachment	−0.11	−0.07	0.16	0.15	0.16	0.16	0.16	0.04						
10. Avoidant Attachment	−0.24 **	0.08	0.17	0.10	0.13	0.21 **	0.06	−0.04	0.47 **					
Health														
11. Physical Well-being at baseline	0.01	−0.12	−0.08	−0.12	−0.17	0.01	−0.06	−0.02	−0.30 ***	−0.29 ***				
12. Physical Well-being at 4 months	0.01	−0.11	−0.09	−0.12	−0.17	0.01	0.03	0.01	−0.23 *	−0.26 **	0.67 ***			
13. Physical Well-being at 8 months	−0.10	−0.08	−0.06	−0.27 *	−0.12	−0.02	−0.18	−0.04	−0.35 ***	−0.28 **	0.74 ***	0.72***		
14. Physical Well-being at 12 months	0.08	−0.06	−0.09	−0.13	−0.09	−0.04	0.03	−0.18	−0.09	−0.13	0.55 ***	0.52 ***	0.49 ***	
Mean (SD)	0.96 (0.59)	1.01 (0.68)	0.29 (0.49)	0.48 (0.48)	1.38 (0.46)	2.10 (0.72)	2.99 (0.45)	1.41 (0.44)	1.90 (0.75)	2.52 (0.67)	3.51 (0.47)	3.45 (0.59)	3.45 (0.53)	3.44 (0.54)
n	134	134	134	134	134	134	134	134	133	133	133	103	94	88

*Note*. * *p* < 0.05. ** *p* < 0.01. *** *p* < 0.001. Data reflect responses of study variables of the partner only.

**Table 3:** Two-level mixed effect models of anxious attachment, affective expression, and communication behavior for patients and partners.

	Positive Joining				Positive Individuating		
	*B*	*SE B*	95% CI		*B*	*SE B*	95% CI
Intercept	0.93 ***	0.05	[0.83, 1.04]	Intercept	1.12 ***	0.06	[1.00, 1.23]
Anxious Attachment	−0.06	0.05	[−0.16, 0.05]	Anxious Attachment	−0.08	0.05	[−0.18, 0.02]
Role	0.02	0.06	[−0.10, 0.14]	Role	−0.11 *	0.05	[−0.21, −0.01]
Role × Anxious Attachment	−0.02	0.08	[−0.18, 0.14]	Role × Anxious Attachment	0.02	0.07	[−0.12, 0.16]
	Hard Negative				Soft Negative		
	*B*	*SE B*	95% CI		*B*	*SE B*	95% CI
Intercept	0.32 ***	0.04	[0.24, 0.40]	Intercept	0.82 ***	0.05	[0.73, 0.92]
Anxious Attachment	0.12 **	0.04	[0.04, 0.21]	Anxious Attachment	0.15 **	0.05	[0.05, 0.25]
Role	−0.02	0.05	[−0.12, 0.09]	Role	−0.33 ***	0.06	[−0.45, −0.21]
Role × Anxious Attachment	−0.01	0.07	[−0.15, 0.12]	Role × Anxious Attachment	−0.05	0.08	[−0.20, 0.11]
	Positive Approach				Positive Avoidance		
	*B*	*SE B*	95% CI		*B*	*SE B*	95% CI
Intercept	3.06 ***	0.04	[2.98, 3.14]	Intercept	1.26 ***	0.03	[1.19, 1.33]
Anxious Attachment	0.03	0.04	[−0.06, 0.11]	Anxious Attachment	0.02	0.04	[−0.05, 0.09]
Role	−0.06	0.05	[−0.16, 0.05]	Role	0.15 **	0.05	[0.05, 0.24]
Role × Anxious Attachment	0.08	0.07	[−0.05, 0.21]	Role × Anxious Attachment	0.00	0.06	[−0.11, 0.12]
	Negative Approach				Negative Avoidance		
	*B*	*SE B*	95% CI		*B*	*SE B*	95% CI
Intercept	1.35 ***	0.04	[1.27, 1.42]	Intercept	1.91 ***	0.06	[1.80, 2.02]
Anxious Attachment	0.11 **	0.04	[0.03, 0.19]	Anxious Attachment	0.04	0.06	[−0.08, 0.16]
Role	0.04	0.05	[−0.05, 0.13]	Role	0.20 **	0.07	[0.06, 0.34]
Role × Anxious Attachment	0.00	0.06	[−0.11, 0.12]	Role × Anxious Attachment	0.14	0.09	[−0.05, 0.32]

*Note.* Anxious attachment was grand-centered. For role, patient was coded as 0 and partner was coded as 1. * *p* < 0.05. ** *p* < 0.01. *** *p* < 0.001.

**Table 4:** Two-level mixed effect models of avoidant attachment, affective expression, and communication behavior for patients and partners.

	Positive Joining				Positive Individuating		
	*B*	*SE B*	95% CI		*B*	*SE B*	95% CI
Intercept	0.92 ***	0.05	[0.82, 1.02]	Intercept	1.11 ***	0.06	[0.99, 1.22]
Avoidant Attachment	−0.24 **	0.07	[−0.39, −0.10]	Avoidant Attachment	−0.17 *	0.07	[−0.31, −0.04]
Role	0.04	0.06	[−0.08, 0.16]	Role	−0.09	0.05	[−0.19, 0.01]
Role × Avoidant Attachment	0.06	0.10	[−0.14, 0.27]	Role × Avoidant Attachment	0.11	0.09	[−0.07, 0.29]
	Hard Negative				Soft Negative		
	*B*	*SE B*	95% CI		*B*	*SE B*	95% CI
Intercept	0.33 ***	0.04	[0.25, 0.41]	Intercept	0.83 ***	0.05	[0.74, 0.93]
Avoidant Attachment	0.19 **	0.06	[0.07, 0.31]	Avoidant Attachment	0.07	0.07	[−0.08, 0.21]
Role	−0.04	0.05	[−0.14, 0.07]	Role	−0.35 ***	0.06	[−0.47, −0.23]
Role × Avoidant Attachment	−0.07	0.09	[−0.24, 0.10]	Role × Avoidant Attachment	0.02	0.10	[−0.18, 0.22]
	Positive Approach				Positive Avoidance		
	*B*	*SE B*	95% CI		*B*	*SE B*	95% CI
Intercept	3.06 ***	0.04	[2.98, 3.14]	Intercept	-	-	-
Avoidant Attachment	−0.00	0.06	[−0.12, 0.12]	Avoidant Attachment	-	-	-
Role	−0.06	0.05	[−0.17, 0.04]	Role	-	-	-
Role × Avoidant Attachment	−0.05	0.09	[−0.21, 0.12]	Role × Avoidant Attachment	-	-	-
	Negative Approach				Negative Avoidance		
	*B*	*SE B*	95% CI		*B*	*SE B*	95% CI
Intercept	1.36 ***	0.04	[1.28, 1.43]	Intercept	1.91 ***	0.06	[1.80, 2.02]
Avoidant Attachment	0.16 **	0.06	[0.05, 0.27]	Avoidant Attachment	0.11	0.08	[−0.06, 0.27]
Role	0.02	0.04	[−0.07, 0.10]	Role	0.18 *	0.07	[0.03, 0.32]
Role × Avoidant Attachment	−0.06	0.08	[−0.20, 0.09]	Role × Avoidant Attachment	0.14	0.12	[−0.09, 0.37]

*Note.* Avoidant attachment was grand-centered. For role, patient was coded as 0 and partner was coded as 1. In this model, positive avoidance could not be calculated due to low variance. * *p* < 0.05. ** *p* < 0.01. *** *p* < 0.001.

## Data Availability

Our data are available upon request pending approvals from our Institutional Review Board. If interested, please e-mail the corresponding author.
